# *Trichinella *inflammatory myopathy: host or parasite strategy?

**DOI:** 10.1186/1756-3305-4-42

**Published:** 2011-03-23

**Authors:** Fabrizo Bruschi, Lorena Chiumiento

**Affiliations:** 1 Department of Experimental Pathology, M.B.I.E., Università di Pisa, 56126 Pisa, Italy

## Abstract

The parasitic nematode *Trichinella *has a special relation with muscle, because of its unique intracellular localization in the skeletal muscle cell, completely devoted in morphology and biochemistry to become the parasite protective niche, otherwise called the *nurse cell*. The long-lasting muscle infection of *Trichinella *exhibits a strong interplay with the host immune response, mainly characterized by a Th2 phenotype.

The aim of this review is to illustrate the role of the Th2 host immune response at the muscle level during trichinellosis in different experimental models, such as knock-out or immuno-modulated mice. In particular, in knock-out mice a crucial role of IL-10 is evident for the regulation of inflammation intensity.

The muscular host immune response to *Trichinella *is partially regulated by the intestinal phase of the parasite which emphasizes the intensity of the following muscle inflammation compared with animals infected by synchronized injections of newborn larvae. In eosinophil-ablated mice such as PHIL and GATA-- animals it was observed that there was an increased NOS2 expression in macrophages, driven by higher IFN-γ release, thus responsible for muscle larva damage.

Besides modulation of the intestinal stage of the infection, using recombinant IL-12, increases the muscular parasite burden delaying adult worm expulsion from the intestine. Furthermore, a Th1 adjuvant of bacterial origin called *Helicobacter pylori *neutrophil activating protein (HP-NAP), administered during the intestinal phase of trichinellosis, alters the Th2 dependent response at muscle level.

All these data from the literature delineate then a mutual adaptation between parasite and host immune response in order to achieve a strategic compromise between two evolutionary forces pointed towards the survival of both species.

## Introduction

The inflammatory myopathies (pathological abnormalities of the muscles) are a group of muscle diseases characterised by inflammation of the muscles or associated tissues, such as for example the blood vessels that supply the muscles themselves. Another term to indicate this kind of pathological process is *myositis*.

The chronic inflammatory process is sustained by the "invading" cells of the immune system of the host, such as neutrophils, eosinophils, activated macrophages and T-lymphocytes that lead to the destruction of muscle tissue, accompanied by weakness and sometimes pain; therefore over time, there could be loss of muscle bulk (atrophy). When phagocytic cells clear necrotic tissue and quiescent satellite cells are activated by growth factors, muscle regeneration occurs, creating in the same tissue, both damage and regeneration.

Inflammatory myopathies are not linked to specific genetic defects, although genetic factors can delineate the predisposition to develop inflammatory myopathies. The cause of an inflammatory myopathy may be unclear for several different reasons. For example, the host immune system turns against its own muscles and damages muscle tissue in an autoimmune response [1]. In other cases, muscle inflammation may be caused by an allergic reaction, cancer or rheumatoid conditions, or following environmental exposure to xenobiotics or drugs, or infection with viruses (influenza, Coxsackie viruses, arboviruses), bacteria (i.e. Lyme disease-related *Borrelia *species) and parasites (*Toxoplasma gondii*, *Trypanosoma cruzi*, *Sarcocystis *spp. among protozoa and *Taenia solium *and *Trichinella *among helminths). All these infectious agents fight part of their combat survival in the muscles. This review deals in particular with myositis in *Trichinella *infection.

This nematode is the etiological agent of trichinellosis, a zoonosis that involves mammals including man, birds and reptiles. *Trichinella *differs from other helminths because its life-cycle which is completed in a single host involves two distinct intracellular habitats, intestinal epithelium and skeletal muscle cell [2].

The myopathy in trichinellosis is defined *remote*, however in experimental trichinellosis in guinea pigs, by analysis of serial sections, Drachman and Tunebay (23) were able to demonstrate larvae, undetected by routine techniques, in a number of myofibers, concluding that pathology is confined to regions in the immediate proximity of the larvae [3]. The myositis in trichinellosis has begun only recently to be elucidated in the fine mechanisms, involving cells of immune system such as T helper (Th) 1 cells, Th2 , eosinophils, macrophages, which are the subjects of the present review. Activation of Th corresponds to different mechanisms and cytokine secretion profiles involving different effector cell acting against various pathogens, such as macrophages and Natural Killer cells in the case of Th1 phenotype or eosinophils, mast cells and IgE secreting B cells when the response is shifted to a Th2 type.

### Myositis during trichinellosis

Among helminths, only *Trichinella *spp. have a fascinating and close relationship with muscle tissue. In fact, after invading the skeletal muscle fibre cells, it adopts a Trojan Horse strategy. This parasite deceives the host muscle cell entering and shaping itself inside its protective niche, otherwise called, *nurse cell *(NC) [2]. Furthermore, *Trichinella *strategy for NC formation involves also satellite cells since they undergo cell division and join to the invaded skeletal muscle fiber in forming the NC [4-6]. Besides the cytoplasm of the NC hosts cells of immunity such as CD4+ and CD8+ T lymphocytes, probably trapped in it [7] or invading [8], before the encapsulation process. These cells coexist within the NC without causing apparent damage to the parasite [7]. This host-helminth parasite relation is unique for the mammalian immune response which tries to combat *Trichinella *inside the NC, establishing a chronic inflammation to the site of infection. *Trichinella *larvae, after entering the skeletal muscle tissue, induce a relevant inflammatory reaction which is responsible for myositis, one of the typical consequences of the parenteral infection phase also evident in humans (see later). Host tissue damage is caused not only directly by the parasite itself, but also indirectly, for the presence of inflammatory cells which produce high levels of reactive oxygen species and other free radicals, after activation [9]. Recently, it has been shown, in fact, that the NC in *Trichinella *infected animals undergoes an oxidative stress process, as revealed by the increased production of glutathione-S-transferase I, as well as of heme-oxygenase I, a typical stress marker, compared to the surrounding muscle fibres. Furthermore, the NC is enriched with lipoperoxydised proteins. This stress response appears to be more evident, in muscles derived from animals infected with an encapsulating species such as *T. spiralis*, than in those from mice infected with the non-encapsulating *Trichinella pseudospiralis*, suggesting a possible correlation with the extent of inflammatory reactions which is lower in the latter case [10].

### Inflammatory response to different *Trichinella *species

Myopathy which is referred to muscle cell changes including loss of its striation and transformation to the NC, depends on *Trichinella *species responsible for infection, in fact in experimentally infected mice with the non-encapsulating species *T. pseudospiralis*, myopathy is more prolonged and diffuse than in *T. spiralis *infected mice but the reasons why this happens are not yet understood [11].

The host response to muscle invasion has been evaluated in animals differing genetically in immune status, focusing both on the extent of the inflammatory response surrounding *T. spiralis *and *T. pseudospiralis *species and the cytokine pattern and the type of T helper cell infiltrating the muscle fibers in experimental infections [7]. The results have shown different levels of cell infiltration in response to *T. spiralis *larvae, depending on the mouse strain. In general, during infection with *T. pseudospiralis*, a lower number of infiltrating cells (seen in BALB/c mice) or total absence of infiltrating cells (seen in CBA/N and nude mice) were assessed as compared to *T. spiralis *infection, confirming previous observations on differences in encapsulated and non-encapsulated species of the genus *Trichinella *[12,13].

Cytokines produced by lymphocytes derived from popliteal lymph nodes of mice infected synchronously by injecting *T. spiralis *newborn larvae (NBL) directly in the leg muscles, demonstrated a typical T helper 2 (Th2) activation pattern [7]. This polarised response was confirmed in humans, where cellular immunity in blood cells was studied during the muscle phase in both *T. spiralis *and *T. britovi *infections [14]. Tissue eosinophilia surrounding the NC-parasite complex can be more easily explained in light of this type 2 cytokine pattern.

As already stated, for a long time, the existence of differences in inflammatory response to the various *Trichinella *species [12] has been shown, but this conclusion was drawn simply by observing microscopically the areas surrounding the parasite. By means of an image analysis based on evaluation of labelled nuclei, it was confirmed not only that the non-encapsulated species (*T. pseudospiralis*) induce a lower inflammatory reaction around the parasites than that observed around encapsulated larvae (*T. spiralis *and *T. britovi*) but also that *T. spiralis *is accompanied by the higher inflammatory response, compared to that induced by *T. britovi *[15]. This result was not related to differences in fertility between adults of the two species examined, and consequently to the different number of larvae that appeared in the muscles [16]. The method used, allowed an evaluation of only the response around each single larva and not the whole inflammatory response in the muscles, dependent on the parasite burden. In addition, this method was able to quantify the reaction in muscle tissues far from the parasite, demonstrating that, even in the extra-capsular areas, the inflammation is lower in muscle tissues infected with non-encapsulated, than in those with encapsulated species. It can then be concluded that the significant inflammation gradient existing between peri-capsular and extra-capsular areas of encapsulated species is not present in non-encapsulated species in which no difference was observed between the two areas, suggesting that in the latter case the parasite is unable to develop a specific chemotactic signal.

These findings are in agreement with Shupe and Stewart (1991) [17], who showed that a crude extract from *T. pseudospiralis *inhibits neutrophil chemotaxis more than that from *T. spiralis*, but in the case of the non-encapsulated species the flow of molecules is not impeded by a thick collagen capsule. Consequently, a higher concentration and wider distribution of the suppressive molecules occur in the infected muscle tissue. In our study, this is confirmed by the reduction of nuclei accumulation in the extracapsular area, indicating a low level of myositis. In addition, a higher chemotactical response of neutrophils from *T. pseudospiralis*-infected mice was observed *in vitro*, compared with that from uninfected or *T. spiralis*-infected mice [17].

Differences in inflammatory response around the NC-parasite complex were observed by classical morphometric methods also between *T. spiralis *and *T. nativa*-infected raccoon dogs. Inflammatory response resulted higher around *T. nativa *[18], however, using this method it was not possible to provide any information about extracapsular areas.

### Regulation of inflammatory response at muscle level

Although the intestinal inflammatory response to *Trichinella *adult worms has been widely investigated, in an attempt to understand the fine mechanisms responsible for worm expulsion (reviewed in [19]), only recently has a direct relationship during the course of *T. spiralis *infection between the intestinal inflammatory response and myositis been observed [20]. Furthermore, the intestinal phase of the parasite influences the following muscle invasion. In fact, mice infected *per os*, the natural route of infection, display an enhanced myositis, mainly represented by an increased number of eosinophils and neutrophils, compared with mice intravenously injected with NBL, where the intestinal phase is absent [21]. The results of these experiments are in contrast with those of Li and Ko (2001) [7], who showed a substantially stronger muscle inflammation in animals injected subcutaneously with NBL than in orally infected mice. Probably, the different results obtained by the two studies are due to different times of infection and inoculum size which was calibrated especially by Fabre *et al. *[21] to have similar numbers of NBL arrived to the muscles in the different ways of infection. However, it has already been shown that the immunological modulation of the intestinal phase of infection regulates the following muscular phase, too. In fact, the administration of recombinant IL-12 during the first day of infection delays worm expulsion and increases fecundity through the reduction of intestinal eosinophils and goblet cells, along with a decreased Th2 phenotype leading to an increased number of encysted muscle larvae, independently on the production of IFN-γ [22].

The immunological response to *T. spiralis *muscle invasion is mainly characterized by a Th2 phenotype, in fact cells collected from cervical lymph nodes of infected mice produce IL-5, IL-10, IL-13 and IFN-γ after stimulation with somatic larval antigens [23] and by the presence of parasite-specific IgG1 and IgE during the chronic infection [24]. The cellular infiltrate surrounding the NC is mainly composed of macrophages, able to invade the cytoplasm of the NC, too [24]. CD4+ T cells, fewer CD8+ T cells, and rare B lymphocytes represent the remaining cell types present in the infiltrate.

In knock-out (KO) mice for the interleukin-10 (IL-10) gene (IL10-/-), the extent of the inflammatory infiltrate around the NC was markedly increased compared with control mice during the acute phase of infection, though the cellular composition remained the same [24,25]. Although histological evidence displayed an increase in cell infiltrate, in IL10 -/- mice, NC viability (integrity), parasite establishment and survival remained unaffected. Furthermore, in IL-10 -/- mice the macrophage infiltrate appeared immunohistochemically to be rich in iNOS or NOS 2 (inducible nitric oxide synthase, an enzyme responsible for the production of nitric oxide) producing cells in contrast with the few stained cells present in control mice [23]. IL-10 probably might reduce inflammation by suppressing the IFN-γ release in lymphocytes recovered from cervical lymph nodes and consequently the NOS 2 expression in macrophages, driving a Th2 regulated muscle inflammation only during the initial development of the parasite. The effect of IL-10 becomes more evident when infected BALB/c mice deficient in STAT 6, a transcription factor responsible for the differentiation of Th2-polarized cells, mounted a moderate inflammation in response to the NC, sustained by lower levels of IL-4, IL-5 and IL-13 (released by lymphocytes derived from cervical lymph nodes) but higher IFN-γ production, compared with control mice. Then, even in the absence of a stabilized Th2 phenotype in response to *Trichinella *infection, IL-10 would modulate the emerging T helper 1 (Th1) activation during the early phase of infection. Adoptive transfer experiments of effector T lymphocytes (Teff) or regulatory T cells (Treg) from wild-type or IL-10 -/- mice in recipient mice deficient in T and B lymphocytes identified the source of IL-10, necessary to suppress myositis, in T eff cells CD4+ CD25- [23]. In addition to IL-10 anti-inflammatory effect, the transforming growth factor beta (TGF-β), involved in the immunosuppression of T eff cells by Treg [26], acts in concert with this cytokine. In fact, IL-10 -/- mice treated with anti-TGF β develop a strong inflammation around the NC-parasite complex with a worm burden significantly reduced compared to control mice [23]. Interestingly, IL 10 -/- mice first infected and then challenged with the synchronous injection of NBL of *T. spiralis *display histologically damaged NC and largely invaded by infiltrating cells [23], suggesting a relation between an increased Th1 response and parasite damage.

### The role of eosinophils in experimental trichinellosis

In the recent paper by Fabre *et al. *(2009) [21] muscle inflammation was studied during *T. spiralis *infection in two models of eosinophil ablation, Δdbl-GATA^_/_ ^and eosinophil peroxidase diphtheria toxin transgenic mice (PHIL). Mice deficient in Δdbl-GATA have a deletion of the high-affinity double GATA site in the GATA-1 promoter and are characterised by a failure of eosinophil differentiation. In fact, GATA-1 is a transcription factor which reprograms immature myeloid cells to three different hematopoietic lineages such as erythroid cells, megakaryocytes, and eosinophils. PHIL mice represent another model of eosinophil ablation, induced with the incorporation of a coding sequence for the diphtheria toxin A chain in the eosinophil peroxidase locus. As expected, in both these mice, infiltrates surrounding NCs completely lack eosinophils which are also absent in blood of infected mice, in contrast to wild-type mice but, surprisingly, *T. spiralis *muscle larvae were recovered in lower numbers, compared to non-genetically modified animals with a reduction of about 60-70% in PHIL mice and 48% in Δdbl-GATA^_/_ ^animals. The lower parasite burden was accompanied by an enhanced Th1 response and downregulated Th2 response. In genetically-ablated eosinophil animal groups, lymph node cell produced increased levels of IFN-γ and decreased IL-4 in *in vitro *culture. As a result of Th1 activation, induced macrophages can produce the enzyme NOS 2 which transforms L-arginine to nitric oxide, responsible for parasite damage. The blocking of this enzyme with specific inhibitors obtained a better larval survival. In mice genetically modified, not only for the function of eosinophil peroxidase gene (PHIL mice) but also for that of IL-10 (double deficient IL-10^_/_^/PHIL mice), a dramatic reduction in the larval burden (93%) compared with mice deficient only in IL-10 was observed. In addition, when these animals were treated with the NOS 2 inhibitor, in both IL-10-/- or IL-10-/-/PHIL mice the lymph node cell produced lower amounts of NO in cultures and in parallel larval survival increased. These results show that muscle larvae are damaged by an immune response driven by Th1 cells which seem to be downregulated by eosinophils. This cell population can therefore play a Janus role of effector or regulatory functions. We could speculate that the parasite induces eosinophilia to protect itself.

**Figure 1:**
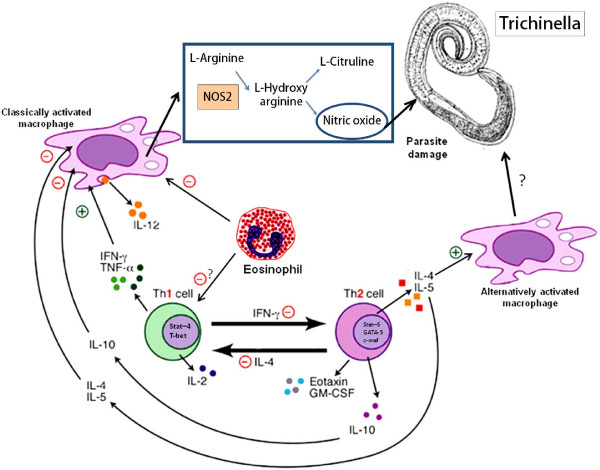
***Trichinella spiralis *elicits in its host a T helper 2 (Th2) cell differentiation that inhibits Th1 cell activation and suppresses classically activated macrophage by IL-4, IL-5 and IL-10 actions**. In particular the Th2 cytokines IL-4 and IL-13 act as sufficient stimuli for the differentiation of alternatively activated macrophages which effects on the parasite, protected by the nurse cell, are not well known. In the genetically manipulated experimental model, the lack of eosinophils can influence, through different mechanisms, the outcoming Th1 activation and promote consequently classical macrophage activation. In these cells the production of nitric oxide (NO) by their NOS2 enzyme provokes parasite damage and influences the trasmission to the next host. + = stimulatory pathway - = inhibitory pathway

A summary of possible interactions between Th1-Th2, eosinophils and macrophages in trichinellosis, according to experiments in KO mice is shown in Figure 1.

It was possible, by up-regulating the Th1 response, using one of the virulence factors of the bacterium *Helicobacter pylori*, the so-called neutrophil activating protein (HP-NAP) [27] to modulate Th2 response in experimental trichinellosis [28]. In the same model, an increase of the inflammatory response was observed as well as a decrease of eosinophils around the NC, in *T. spiralis *infected mice, confirming the importance of Th2 and perhaps of Treg in regulating tissue eosinophilia (Chiumiento et al., submitted). Furthermore, the inflammatory infiltrate surrounding the parasite of infected untreated animals was characterized by increased immunostaining of arginase I which is, in addition to the chitinase-like molecule YM1, a marker of alternatively activated macrophages (AAM) by the Th2 response, compared with infected treated animals (unpublished results). This would suggest a higher Th2 activation in infected untreated animals. The formation of AAM and induction of the molecule YM1 have been shown in both mice and guinea pigs infected with *T. spiralis *[reviewed in 29], however the role of these cells in trichinellosis is not yet defined.

The Th17 cells were studied during trichinellosis with special emphasis on intestinal immunity, in fact they control the intestinal smooth muscle hypercontractility but little is known as regards muscle inflammation and further research is needed on this issue [30].

### Myositis in human trichinellosis

The parenteral or muscular phase in humans is associated with inflammatory and allergic responses caused by invasion of the skeletal muscle cells by the migrating larvae. This invasion, as already stated can damage the muscle cells, either directly or indirectly stimulating the infiltration of inflammatory cells, primarily eosinophils. A correlation between the eosinophil levels and serum muscle enzymes such as lactate dehydrogenase and creatine phosphokinase, has been observed in trichinellosis patients, suggesting that muscle damage may be mediated indirectly by these activated granulocytes [31]. But muscle tissue could be damaged also by immunopathological processes. In fact, in late trichinellosis occurring several years after infection, the presence in the sera of skeletal muscle specific antibodies has been observed, recognizing 28 and 41 kDa proteins in this tissue extract [32].

## Conclusions

Information derived from the studies on *Trichinella*-induced muscle inflammation provide new perspectives of the host-parasite relationship. According to results obtained from experiments in genetically eosinophil-ablated mice, simulating a shift towards a Th1 immune response, larvae are damaged, compromising parasite transmission to the next host. For this reason it appears beneficial for the parasite to elicit in its host a Th2 response leading to tissue eosinophilia, during the muscle stage of infection. However, this host response is not only protective for the parasite but also for the ameliorate myositis which is more diffuse in Th1 up-regulated conditions.

The muscle phase of *Trichinella *infection is the result of host and parasite mechanisms which delineate the subsequent myositis in a crossroad of different intents, where parasite establishment collides with host survival and one element limits the other thus to accomplish the survival of both species.

## Competing interests

The authors declare that they have no competing interests.

## Authors' contributions

FB and LC contributed equally to the elaboration of the manuscript, drafting the manuscript and designing Figure 1. All authors read and approved the final manuscript.
